# Applying the Inverse Efficiency Score to Visual–Motor Task for Studying Speed-Accuracy Performance While Aging

**DOI:** 10.3389/fnagi.2020.574401

**Published:** 2020-12-09

**Authors:** Yauhen Statsenko, Tetiana Habuza, Klaus Neidl-Van Gorkom, Nazar Zaki, Taleb M. Almansoori

**Affiliations:** ^1^Radiology Department of College of Medicine and Health Sciences, United Arab Emirates University, Al Ain, United Arab Emirates; ^2^Department of Computer Science and Software Engineering of College of Information Technology, United Arab Emirates University, Al Ain, United Arab Emirates

**Keywords:** aging, cognitive decline, speed-accuracy trade-off, decision making, error, machine learning, regression model, gender

## Abstract

**Background:** The current study examines the relationship between speed and accuracy of performance in a reaction time setting and explores the informative value of the inverse efficiency score (IES) regarding the possibility to reflect age-related cognitive changes.

**Objectives:** To study the characteristics of speed and accuracy while performing psychophysiological tests throughout the lifespan; to examine the speed-accuracy ratio in age groups and to apply IES to discriminative visual-motor reaction task; and to figure out the predictive potential of psychophysiological tests to identify IES values.

**Methods:** We utilize nonparametric statistical tests, regression analysis, and supervised machine learning methods.

**Results and Conclusion:** The examinees under 20 and over 60 years of age share one tendency regarding the speed-accuracy ratio without speed-accuracy trade-off. Both at the time of active developmental changes in adolescence and during ongoing atrophic changes in elderly there is a tendency toward a rise of the number of mistakes while slowing the reaction. In the age range from 20 to 60 the relationship between the speed and accuracy is opposite and speed-accuracy trade-off is present. In our battery, complex visual-motor reaction is the only test with the significant negative association between reaction time and error rate in the subcohort of young and midlife adults taken together. On average, women perform more slowly and accurately than men in the speed-accuracy task, however most of the gender-related differences are insignificant. Using results of other psychophysiological tests, we predicted IES values for the visual-motor reaction with high accuracy (*R*^2^ = 0.77 ± 0.08; mean absolute error / IES range = 3.37%). The regression model shows the best performance in the cognitively preserved population groups of young and middle-aged adults (20–60 years). Because of the individual rate of neurodevelopment in youth and cognitive decline in the elderly, the regression model for these subcohorts has a low predictive performance. IES accounts for different cognitive subdomains and may reflect their disproportional changes throughout the lifespan. This encourages us to proceed to explore the combination of executive functioning and psychophysiological test results utilizing machine learning models. The latter can be designed as a reliable computer-aided detector of cognitive changes at early stages.

## 1. Introduction

A speed-accuracy trade-off (SAT) in behavioral decisions is a physiological phenomenon that accounts for the adjustment of species to living conditions. SAT is a feature of the individual's psychophysiological status that can dynamically change in a certain range of values under certain conditions, otherwise, it remains stable (Wang et al., 2018). Individuals differ in cognitive styles, and the individual traits of SAT may account for cognitive performance (Jones et al., 2020).

On average, accurate decisions require more time, while fast decisions are usually less accurate. However, in some circumstances, both variants could be an option. Speed-accuracy tactics are known to vary consistently and show a degree of flexibility during task fulfillment. Such individual flexibility in speed-accuracy tactics is likely to be advantageous for animals exposed to fluctuating environments, such as changes in predation threat (Wang et al., 2018). According to the cognitive styles hypothesis, individuals with consistently low levels of activity and higher sensitivity to risk may be expected to take more time but make more accurate decisions than individuals that are more active and less sensitive to risk. Based on this, one can argue that SATs underlie *interindividual differences in cognition* (Jones et al., 2020). This implies that SATs may account for *cognitive styles* which we can discriminate by testing the individuals and estimating their trade-offs. In any test, the examinee has a substantial degree of control over speed or accuracy at which s/he chooses to operate. Yet, the individuals act within the boundaries of personal performance limitations. They may adjust these boundaries continuously depending on error feedback (Fitts, 1966).

The clinical necessity to develop the aforementioned cognitive theories and to measure SAT comes out of the practical importance to estimate psychophysiological status by providing clearly stated metrics of its current condition and changes.

### 1.1. Speed and Accuracy Performance During Development and Atrophy

#### 1.1.1. The Impact of Neurodevelopment on Speed and Accuracy

Even within a narrow 5–7 years of age participants group, researchers found that *older children are faster and more accurate* (Torpey et al., 2012). In this study, associations between the gender of the child and the behavioral modes suggested that girls were more cautious than boys as found in other age groups.

#### 1.1.2. Age-Related Changes in Speed and Accuracy

Many findings indicate a consistent pattern of increased reaction time (RT) with age in both genders in a variety of tasks. Fozard et al. (1994) conducted a longitudinal study of aging with adult volunteers 17–104 years of age. They estimated a consistent slowing down of auditory reaction which starts at about the age of 20 and increases at a rate of approximately 0.5 ms/yr for simple reaction and 1.6 ms/yr for the disjunctive reaction. In this observation the number of errors also increased throughout the lifespan, *making a tradeoff of accuracy for faster responses unlikely*.

Facts suggest a possible *impairment of response inhibition* in old age. As an outcome of this, the elderly may experience difficulty with sustaining attention to the stimuli exclusively. Presumably, this is caused by a decreased ability to inhibit irrelevant thoughts (Arbuckle and Gold, 1993). The individual features and the age-related changes in visual-attention control may also account for different cognitive performance. This may happen at least for two reasons. These are either a decline in overall bottom-up sensory input, or decrease in the differentiation of top-down goal-directed representations (Heitz and Engle, 2007; Li et al., 2013).

### 1.2. A Statistical Approach to the Problem

A number of studies have developed various models to account for the speed-accuracy payoff (Thompson, 2007). This is based on the fact that a part of the choice-reaction time is devoted to decision making and processes that are analogous to those employed in the simple sensorimotor reaction (Fitts, 1966). *The random walk model* initially suggested by Stone (Stone, 1960) is one of the simplest to make time series forecasting. It possesses enough structure to predict accuracy and latency results. The random walk model for two choice reaction times assumes that there is an information accumulation over the course of perceptual decision-making. In other words, evidence in favor of these responses is accumulated gradually over time until the evidence favoring one over the other exceeds some preset criterion, at which time the favored response is emitted (Ashby, 1983). The random walk model is a sequential model, i.e., it assumes the incremental evidence accumulation, i.e., faster responses entail less accumulated evidence, and hence less informed decisions (Heitz, 2014). As an alternative to this, Ollman explained test performance with *a mixture model*. It is a mixture of dichotomous states: fast guesses and slow controlled decisions. The most obvious is that error RT is in average faster than correct RT (Ollman, 1966). The fact that error RT is sometimes faster and sometimes slower than correct RT is problematic for mixture models (Heitz, 2014).

The correct-to-wrong responses ratio reflects the accuracy and accounts for decision processes and the level of education of the examinee. The reaction time (RT) length is determined by cognitive processes and by an individual's use of the feedback information. However, the performance metrics (e.g., RT, ER) may deteriorate if the examinee is set for speed vs. accuracy, or vice versa (Fitts, 1966). The reason for this lies in the ambiguous instruction to maintain both high accuracy and fast RT (Heitz, 2014). For natural reasons emphasis on speed decreases mean reaction time but increases errors. EEG and fMRI studies evidenced that SAT manipulations affect more than decision process (particularly, decision threshold), they alter decision post-processing as well (Nieuwenhuis et al., 2001; Bogacz et al., 2006; Heitz, 2014).

Formerly, authors tried to compare velocity and accuracy characteristics of test performance when subjects are set for speed or correctness. They figured out that error rate may become very small in circumstances where the penalty for errors is sufficiently high, but it never becomes zero as long as the speed of response is given any importance at all (Hick, 1952; Hyman, 1953). Howell and Kreidler (1963) carried out a true SAT experiment in which different groups of participants were asked to favor fast or accurate over fast and accurate responding. With this methodology one can estimate the time cost of an error. SAT is an exact answer to the issue of the time cost of an error. Fitts (1966) pointed out that when the payoff for speed is reasonably high it may induce errors at any level of intelligence or attention. Hence, the occurrence of errors should not be considered as a qualitative change in performance. In such a way, the attempts to induce either “errorless” or accelerated performance are low-informative and their interpretation seems to be challenging.

### 1.3. Speed-Accuracy Estimates Inside Standard Batteries of Psychophysiological and EF Tests

Psychophysiological tests (PT) describe cognitive functioning in terms of domains of functioning. As the cognitive functions are closely linked, it is a challenging task to interpret the changes that may account for mutual compensation (like, SAT). The compensation accounts for the different rate of decline within different cognitive domains. Worsening of diverse performance metrics may start at any age and progress independently. For example, playing tennis preserves high accuracy in coincidence timing performance (Lobjois et al., 2006). Strategic compensation may allow older adults to execute *decision making* at high levels of accuracy (Fechner et al., 2019). The age-related changes in speed and accuracy performance are common results of cognitive retardation (Thapar et al., 2003), however the ratio between them is not studied well-throughout the lifespan.

Clinically, cognitive functioning (e.g., inhibition) is often assessed with the *Stroop color-naming task* different versions of which match the issue of the SAT. In a classical variant of the test, the individual has to inhibit an automatic reading response and to produce the more effortful color-naming task. *The extra time* required to name colors in the interference task compared to the control task (B-A) represents the interference effect (*interference score*). In parallel to this, the *error scores* are recorded. The dualism of executive functions provoked researchers to elaborate a new neuropsychological test that would assess concurrently both inhibition and switching. Such was *Stroop switching test*. Additionally to the classic interference condition (the situation when the word meaning and the ink color doesn't fit), it included switching in-between tasks. The possible clinical application of the idea is to reveal early executive dysfunction that remains undetected with standard neuropsychological tests. So, as opposed to other tests, the compensation strategies do not hinder the early stages of executive dysfunctioning in Stroop switching test. By following the same pipeline, Belghali and Decker (2019) created a modified Stroop switching test version named Stroop switching card test. It encompasses additional conditions and metrics to appraise some more cognitive features so that the global and local variables of the novel test (e.g., the total number of errors performed) reflect the overall individual's executive functioning. The global metrics of performance in the test are the overall time spent on the test and the number of errors done.

(1) $$\begin{array}{l} IES=\frac{TIME}{1-ERROR} \end{array}$$

To study SAT with the tests, researchers combine speed and accuracy into a single dependent variable called the inverse efficiency score (IES). The variable derives from the mean RT and ER. From Equation (1), IES is expressed in milliseconds as well as RT, however, it indicates roughly the time spent for correct responses. When there is a trade-off between speed and accuracy, the IES effect will compensate for the differences in the percentage of incorrect responses.

## 2. Objectives

To get insight as to whether speed-accuracy ratio can serve as a potential biomarker of individual cognitive status while aging we address the following sub-objectives:

1. To study the characteristics of speed and accuracy while performing psychophysiological tests throughout the lifespan.
2. To study the association of the speed-accuracy ratios with the age and cognitive subdomains.
3. To examine the speed-accuracy ratio in age groups and with regard to the gender.
4. To figure out the predictive potential of PTs to identify the values of inverse efficiency scores.

## 3. Materials and Methods

### 3.1. The Used Battery of Tests

The battery of neurophysiological tasks we used fits the idea of our study: it contains PTs with both accuracy and time performance metrics. It has a well-considered structure and reflects a set of cognitive subdomains that underlie goal-targeted behavior. The examinees were paid neither for taking part in the study nor for the achieved results (e.g., good timing or error-less performance) otherwise this would be a limiting non-physiological factor that could spoil outcomes of the research. Structurally, the dataset consists of a list of deidentified subject records, a patient per row, and stored in the comma-separated value format files.

### 3.2. “Psychophysiological Outcomes of Brain Atrophy” Dataset

The dataset is named after the title of the project Psychophysiological Outcomes of Brain Atrophy (POBA). POBA consists of about 100 features reflecting the overall psychophysiological status of examinees. The battery of tests covers diverse aspects of cognitive functioning, both high-level and basic-level ones. It includes 231 cases of MRI examination and PT of people of different ages (4–83 yo). Written patient's consent or parental consent with assent from minors for being tested and scanned was obtained in each case. All the examinees are either patients who suffer from periodic headaches and are anxious about having organic brain pathology or healthy participants examined at the beginning of their professional sports career. The exclusion criteria were as follows: organic brain pathology, mental disorders, injury to the head. The dataset is provided on demand (See section 7). The following tests' form POBA dataset:

*1. Simple visual-motor reaction (SVMR)*. SVMR is the test with the only type of stimulus requiring one and the same response. RT, deemed as the time elapsing between the onset of the stimulus and the initiation of the response, is the major dependent variable. SVMR_mean (ms) is the mean value calculated out of over 30 (SVMR_trialsNo) subsequent episodes of testing with unequal intervals of time between them. SVMR_mean (ms) is the value calculated out of over 30 subsequent episodes of testing (SVMR_trialsNo) with unequal intervals of time between them.

The median (SVMR_median) and the mode (SVMR_mode) values also describe the sample. The measures of how the length of the reaction are scattered in time are the standard deviation (SVMR_variance), the kurtosis (SVMR_kurtosis), the asymmetry (SVMR_ass), the quartile values (SVMR_q25, SVMR_q75), and the half-size interquartile range (SVMR_(q75-q25)/2).

From the physiologic point of view, they may serve as markers of how stable the reaction is in time. Another dependent variable is the number of mistakes made by the examinee (SVMR_mistakes). The mistakes fall into two categories. These are 1. missing the targeted events (SVMR_passes) and 2. preliminary responding (SVMR_falstart). Another dependent variable derived from the test is the Whipple's accuracy coefficient (SVMR_acc_coef). It shows the ratio of errors to correct responses. The lower the indicator is the higher is the performance accuracy. It reflects how stable the attention is and how balanced nervous processes are. In other words, it is a marker of the balance of the excitatory and inhibitory processes.

To assess the sensorimotor response the system also calculates the following indices by Loskutova: system functional level (SVMR_sfl), the reaction stability (SVMR_rs), and the functional ability level (SVMR_fal) (TD, 1975). SVMR_sfl showes the current functional state of the central nervous system at the time of examination, including the rise up of the fatigue. SVMR_rs reflects the stability of the nervous system functioning. The bigger it is, the less the individual's test results are scattered in the time length. The functional ability level (SVMR_fal) is the most valuable index. It reveals the ability of the subject to form a functional system adequate to the task and sustain it for a long-time period. This means, the variable describes the capacity to adjust.

With SVMR time, one can assess the mobility of nervous processes: the shorter RT is, the higher the more mobile the nervous processes are. The variance of the variable (SVMR_variance) depicts the balance of the processes, i.e., the smaller the standard deviation is, the more balanced the nervous system is. SVMR_mean value under 177 ms accounts for the pronounced mobility of nervous processes. Its value of 177–200 ms is a characteristic of a mobile type of nervous processes. With 200–210 ms SVMR_mean length the average type of nervous processes is diagnosed. SVMR_mean value of 210–233 ms is typical for the inertia of nervous processes. Finally, if the reaction lasts more than 233 ms, the pronounced inertia of nervous processes is reported.

Studying the dynamics of RT indicators throughout a day is a way to estimate the strength of the nervous system. With a strong (steady, balanced) nervous system, RT does not change significantly during the day and within the framework of one examination.

*2. Complex visual-motor reaction (CVMR)* is a variant of choice reaction test. A visual stimulus is presented to a subject whose task is to make a motor response quickly and accurately. We used the go/no-go test in which the examinee is asked to push the response button at green indicator light. In contrast, when the indicator lights up with red color no motor response is required. The system records the time elapsed between the onset of a stimulus and a response to. CVMR_mean is the mean length of response time calculated after 30 subsequent presentations of the triggering stimulus. CVMR_mistakes is the number of the fault responses. In the data analysis we use a derivative variable called error rate (ER) (see Equation 3). As in CVMR, we implemented the error rate derivative variables in other tests as well (e.g., SVMR, etc.). There is one more type of the mistakes in the complex reaction compared to the simple one. It is a false reaction to the triggering stimulus of the wrong color, i.e., the responding to the red splash rather than to the green one (CVMR_false_reaction). Similarly to the simple reaction, the complex one also has such dependent variables as CVMR_median, CVMR_mode, CVMR_variance, CVMR_kurtosis, CVMR_ass, CVMR_q25, CVMR_q75, CVMR_passes, CVMR_falstart, CVMR_acc_coef.

Decision-making time (DMT) is the difference between the length of the simple visual-motor reaction and the complex one (see Equation 2). DMT reflects the time cost of the response selection. At the time of making the choice the individual affiliates the cognitive subdomains of switching and inhibition, i.e., the person inhibits prepotent responses and shifts between tasks.

*3. Attention study technique (AST)* is the test in which the examinee is asked to respond to visual stimuli that flick one after another 30 times in diverse parts of the computer screen. Instead of targeting a single point like in the simple visual-motor reaction, the participant is to concentrate attention on the entire PC screen and respond as soon as possible by allocating the same motor reaction as recently.

Reasonably, the dependent variables of AST are similar to that ones in the simple motor-visual reaction time. These are RT (AST_mean, AST_median, AST_mode), the standard deviation (AST_variance), the kurtosis (AST_kurtosis), the asymmetry (AST_ass), the total number of errors with the number of missed stimuli and preliminary responding inside of them (AST_mistakes, AST_delays, and AST_falstart correspondently), the Whipple's accuracy coefficient (AST_acc_coef), and the indices by Loskutova the system functional level (AST_sfl), the reaction stability (AST_rs), the functional ability level (AST_fal).

Additionally, the system provides two estimators that are specific to AST test. These are the attention stability (AST_stability) and the concentration of attention (AST_concentration).

**Figure 1:**
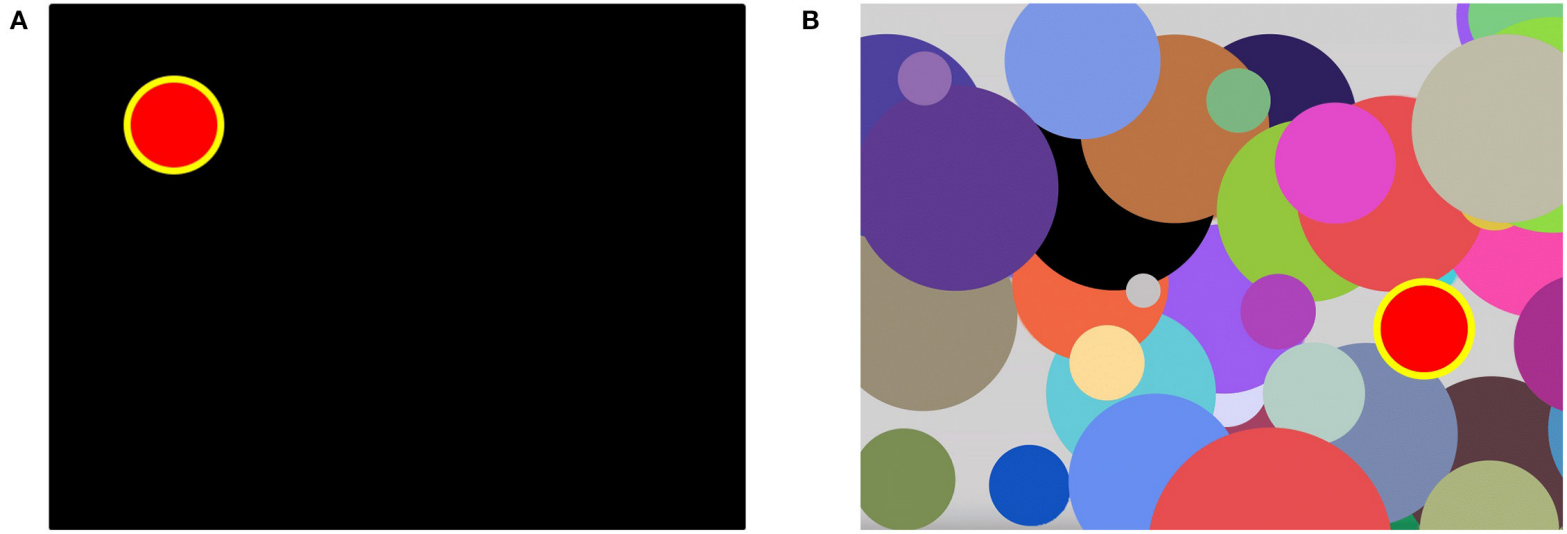
The appearance of the computer screen while testing with attention study technique **(A)** and with interference resilience technique **(B)**. In **(A)**, the stimuli appear sequentially one after another in different parts of the screen. In **(B)**, the interfering visual stimuli pad the screen and complicate the response to specific triggering stimuli.

*4. Interference resilience technique (IRT)* is a bit more complex since the triggering stimuli are obscured with additional interfering objects that appear on the PC screen overlapping the targeted ones (see Figure 1). As the task paradigm is close to the attention study technique, the list of the dependent variables is almost similar.

It includes RT (IRT_mean, IRT_median, IRT_mode), the standard deviation (IRT_variance), the kurtosis (IRT_kurtosis), the asymmetry (IRT_ass), the total number of errors with the number of missed stimuli and preliminary responding inside of them (IRT_mistakes, IRT_delays, and IRT_falstart correspondently), the Whipple's accuracy coefficient (IRT_acc_coef), and the indices by Loskutova the system functional level (IRT_sfl), the reaction stability (IRT_rs), the functional ability level (IRT_fal).

In analogy to DMT, we calculate the time delay in responding to the targeted stimulus because of visual interfering objects (TRVI) as in Formula 4 by substituting of the mean RT without interfering objects (AST_mean) from the study technique with them (IRT_mean).

**Figure 2:**
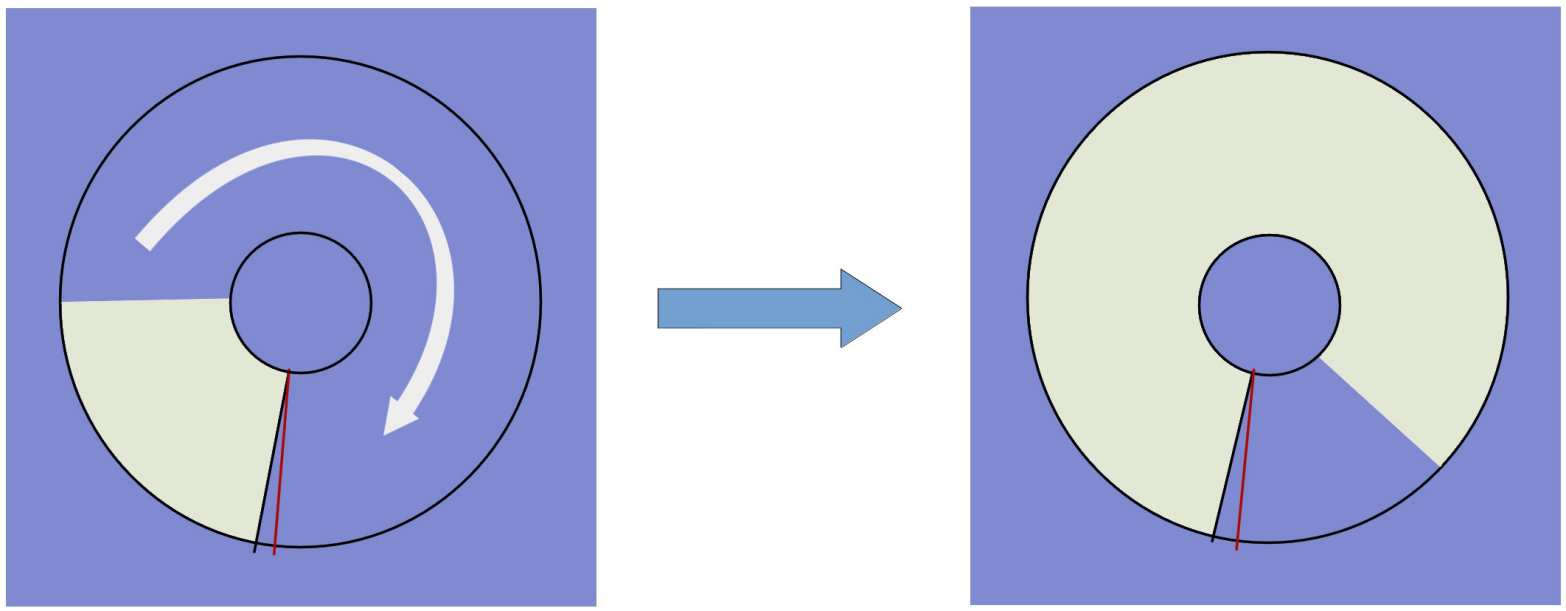
Testing reaction to a moving object: the circle is gradually colored yellow clockwise. The examinee is supposed to respond at the red finishing mark.

*5. Reaction to a moving object technique (RMO)* reflects either the balance of two opposite processes in the central nervous system (e.g., excitation and inhibition) or the predominance of any of them. At the time of the task a circle appears on the screen with two marks of red and green color arranged radially. It is gradually filled quickly with yellow color, from some starting point to the finishing line in a clockwise direction, like, in Figure 2.

The examinee should respond when the yellow sector passes through the red finishing line. There is a total number of over 30 trials (RMO_trialsNo). The result is processed as a mean value (RMO_mean) of positive (the time delays) and negative values (the premature responses). When the RMO_mean value is negative, it indicates the predominance of excitation of the central nervous system. If being positive, RMO_mean reveals the predominance of inhibition of the central nervous system. The tester counts the number of responses that were accurate in time (RMO_acc), the delayed ones (RMO_delays), and the fault starts (RMO_falstart). Also, the application sums up the time of them (RMO_positiveSum, RMO_delaysTotalTime, RMO_falstartTotalTime). All the trials disregarding their accuracy pertain to any of two classes, i.e., the “positive” trials when the response comes at any time after the targeted event and the “negative” ones when the response comes before the event. There are summary counters for the number of positive trials (RMO_positiveCount) and the number of negative ones (RMO_negativeCount).

Like the tests mentioned above, RMO also has such dependent variables as RMO_variance, RMO_median, RMO_mode, RMO_ass, RMO_kurtosis, RMO_q25, RMO_q75. Additionally, there are such metrics as entropy (RMO_entropy), the number of responses

*6. Wrist dynamometry* is a way of evaluating the maximum muscular strength of the right (*WDR_MMS*) and the left hands (*WDL_MMS*). *Asymmetry coefficient (AC)* is calculated from Equation (5).

We calculate the inverse efficiency scores (SVMR_IES, CVMR_IES, AST_IES, IRT_IES) as Formulas 6-9 state.

(2) $$\begin{array}{l} DMT=CVMR\text{\_}mean-SVMR\text{\_}mean \end{array}$$

(3) $$\begin{array}{l} CVMR\text{\_}error\text{\_}rate=\frac{CVMT\text{\_}mistakes}{CVMT\text{\_}trials} \end{array}$$

(4) $$\begin{array}{l} TRVI=IRT\text{\_}mean-AST\text{\_}mean \end{array}$$

(5) $$\begin{array}{l} AC=\frac{WDR\text{\_}MMS}{WDL\text{\_}MMS} \end{array}$$

(6) $$\begin{array}{l} SVMR\text{\_}IES=\frac{SVMR\text{\_}mean}{1-SVMR\text{\_}error\text{\_}rate} \end{array}$$

(7) $$\begin{array}{l} CVMR\text{\_}IES=\frac{CVMR\text{\_}mean}{1-CVMR\text{\_}error\text{\_}rate} \end{array}$$

(8) $$\begin{array}{l} AST\text{\_}IES=\frac{AST\text{\_}mean}{1-AST\text{\_}error\text{\_}rate} \end{array}$$

(9) $$\begin{array}{l} IRT\text{\_}IES=\frac{IRT\text{\_}mean}{1-IRT\text{\_}error\text{\_}rate} \end{array}$$

### 3.3. Age Groups

For the comparison of age cohorts, we added attribute “Groups” to the POBA dataset. The range of years corresponding to age groups is as follow: Adolescent age ∈ [0, 20), Young adults age ∈ [20, 40), Midlife adults age ∈ [40, 60) and Older adults age is ≥ 60.

### 3.4. The Methodology of the Study

*To address the first objective*, we used both the descriptive statistics and the machine learning approach.

As the variables of our dataset had non-normal distribution, we utilized non-parametric tests for the analysis. In age groups, the relationships between continuous features were assessed with Kruskal-Wallis test.

Also we analyzed the charts that describe age-related changes of the mean RT and accuracy. To build the trendlines we used non-linear Locally Weighted Scatterplot Smoothing (LOEWSS), which is a non-parametric regression method that combines multiple regression models in a k-nearest-neighbor-based meta-model.

*To address the second objective* we proposed new psychophysiological indices equivalent to inverse efficiency score in EF tests. As the paradigm of the psychophysiological tests we worked with fitted the idea of SAT, we came up with the indices equivalent to the inverse efficiency score in EF tests. These are *SVMR_IES, CVMR_IES, AST_IES*, and *IRT_IES* (see Formulas 6–9). RMO test results include variables that estimate time and mistakes done. However, in this test, the time variables are the additional estimators of the reaction accuracy (e.g., the delayed reaction or the proactive one) rather than RT. So, the test doesn't fit the concept of SAT, and it cannot provide researchers with an efficiency score.

*In the first part* of objective number two, we inspected possible associations between the age and the newly proposed scores. For this, we built the ordinary least squares (OLS) regression trendlines with a 95% confidence interval. By using Ridge regression we approximated the parameters of the model (e.g., the slope and the intercept) with the polynomial function of degree one and assessed the performance of the models.

To compare the lifelong dynamics of the IES scores for different tests (e.g., CVMR, SVMR, AST, IRT) we tested the linear models for statistically significant differences between the slopes. For this we used statistical hypothesis tests, specifically *t*-test.

To improve the reproducibility and interpretability of the age-related trends we also did sensitivity analyses, in which the RT and error outliers were removed.

*Working on the second part* of objective two, we assessed the relationship between age, tests results that reflect diverse cognitive subdomains and the proposed scores. To do so, we calculated Spearman rank correlation coefficients and checked the associations of variables for the significancy.

*To address the third objective* we inspected possible associations between the mean RT and the overall number of mistakes in age and gender groups. We analyzed the OLS regression trendlines with a 95% confidence interval.

We studied whether the age and gender affected the impact of the RT change on the error rate, i.e., if there is an interaction effect. For this we tested our data for statistically significant differences between the slopes.

*The second part of the third objective* was to find, whether the gender influences the dynamics of age-related changes in RT, efficiency score, mistakes. The statistical approach to this objective was the same as mentioned above. For the gender comparison, we removed outliers from the cohorts of females and males separately and then plotted the trendlines. We assessed the relationships between continuous features with Mann-Whitney *U*-test.

*In the fourth objective* we figured out if IES provides a summary of the findings obtained while testing individuals. This could give the insight to what extent the novel inverse efficiency score reflects the overall psychophysiological status (PS).

To evaluate the prediction potential of the PS estimates to reflect the *CVMR_IES* value, we used the following predictors: “DMT,” “SVMR_mean,” “SVMR_mistakes,” “AST_mean,” “AST_mistakes,” “IRT_mean,” “IRT_mistakes,” “TRVI,” “RMO_mean,” “RMO_mistakes,” “WDL_MMS,” “WDR_MMS,” “AC,” “age.”

For addressing the objectives of the study we utilized supervised machine learning methods, namely regression models. We did this because the predicted values were continuous. Doing this, we used a common approach to data analysis. First, we chose the set of predictors and did a data standardization (removing the mean and scaling to unit variance) as usually required by many machine learning estimators. Then the data were shuffled and fed to the ML regression models in a 10-fold cross-validation manner. In this study, we used such regression algorithms as Lasso, Support Vector Regression with radial basis function kernel, K-nearest neighbors, Gradient Boosting, AdaBoost, and Random Forest. To evaluate the performance of regressors, mean absolute error (MAE), root mean squared error (RMSE), and coefficient of determination (*R*^2^) performance metrics were used.

### 3.5. Hardware and Software Used

All the experiments were conducted with the Linux Ubuntu 18.04 workstation with 24 CPU cores and two NVIDIA GeForce GTX 1080 Ti GPU with 11 GB GDDR5X memory each using programming language Python, and its libraries for Data Processing, ML and Data visualization, such as scikit-learn, NumPy, Pandas, Matplotlib, Seaborn, Plotly.

## 4. Results

### 4.1. The Characteristics of Speed and Accuracy While Performing PT

Table 1 presents the results of testing between-groups differences in psychophysiological variables that reflect reaction speed, accuracy, their ratio, and the information speed processing (e.g., DMT, TRVI). Both DMT and TRVI are the time of inhibition of an automatized action and task switching in the standardized tests. We assume that adolescents experience more difficulties with the concentration of attention, and this impacts the performance of the attention-related tasks, particularly *AST* and *IRT*.

**Table 1:** Tests performance in age groups.

**Variable**	**Total**		**Adolescent**	**Young adults**	**Midlife adults**	**Older adults**	***p*-value**
	**Median**	**IQR**					
N	231		48 (20.78%)	64 (27.71%)	64 27.71%)	55 (23.81%)	
Female	134 (58.01%)		19 (39.58%)^*^	36 (56.25%)	39 (60.94%)	40 (72.73%)^*^	**<0.0078**
Male	97 (41.99%)		29 (60.42%)^*^	28 (43.75%)	25 (39.06%)	15 (27.27%)^*^	
Age	40.96	24.87–59.76	11.52 ± 3.37^*^	29.93 ± 5.09^*^	49.9 ± 6.09^*^	68.09 ± 6.61^*^	**<0.001**
DMT	93.84	63.6–122.43	63.08 ± 52.97^*^	99.88 ± 48.64	96.32 ± 51.65	101.97 ± 57.81	**<0.0006**
TRVI	61.9	34.7–96.0	59.85 ± 60.46	47.0 ± 39.25^*^	66.55 ± 45.34	81.4 ± 74.18	0.148
AC	1.08	1.01–1.19	1.13 ± 0.25^*^	1.06 ± 0.14	1.07 ± 0.2	1.09 ± 0.17	**<0.0079**
SVMR_mean	245.52	219.63–285.83	263.88 ± 70.91^*^	215.22 ± 28.92^*^	242.02 ± 55.48	282.63 ± 53.75^*^	**<0.001**
CVMR_mean	350.75	307.45–395.57	350.82 ± 107.74	320.24 ± 56.55^*^	354.58 ± 65.15	386.15 ± 71.9^*^	**<0.001**
AST_mean	343.4	311.05–412.35	343.7 ± 58.96	309.95 ± 46.58^*^	339.9 ± 61.86	416.0 ± 60.93^*^	**<0.001**
IRT_mean	413.8	368.15–471.85	405.25 ± 84.98	369.1 ± 46.63^*^	417.6 ± 70.65	473.8 ± 75.08^*^	**<0.001**
RMO_mean	7.6	−18.5–31.35	−0.7 ± 69.28	3.6 ± 54.25	22.8 ± 104.22^*^	8.9 ± 75.59	**<0.0065**
SVMR_variance	57.7	41.09–80.82	63.7 ± 73.36	42.86 ± 22.39^*^	56.64 ± 36.54	67.45 ± 42.92^*^	**<0.001**
CVMR_variance	91.81	70.7–118.64	97.72 ± 94.58	79.53 ± 80.43^*^	87.19 ± 30.46	117.08 ± 74.86^*^	**<0.001**
AST_variance	72	53.55–115.6	70.85 ± 57.47	56.65 ± 38.28^*^	74.4 ± 45.85	112.2 ± 62.13^*^	**<0.001**
IRT_variance	103.5	76.75—156.85	133.8 ± 78.28^*^	78.7 ± 40.12^*^	94.95 ± 49.33	150.3 ± 70.46^*^	**<0.001**
RMO_variance	140.3	84.7–224.35	142.1 ± 103.5	101.4 ± 67.33^*^	130.35 ± 93.83	216.7 ± 105.18^*^	**<0.001**
SVMR_mistakes	0	0.0–2.0	1.5 ± 3.83^*^	0.0 ± 1.32^*^	0.0 ± 1.11^*^	1.0 ± 1.54^*^	**<0.001**
CVMR_mistakes	2	1.0–4.0	3.0 ± 2.45^*^	2.0 ± 2.81^*^	2.0 ± 1.75^*^	3.0 ± 2.26^*^	**<0.0003**
AST_mistakes	1	0.0–2.0	0.0 ± 2.09	0.0 ± 1.16^*^	1.0 ± 2.28	2.0 ± 3.33^*^	**<0.001**
IRT_mistakes	3	1.0—6.0	4.0 ± 3.21	2.0 ± 2.35^*^	3.0 ± 3.74	6.0 ± 4.09^*^	**<0.001**
RMO_mistakes	22	18.0–24.0	20.5 ± 5.22	18.5 ± 4.14^*^	22.0 ± 3.82^*^	24.0 ± 3.34^*^	**<0.001**
SVMR_IES	253.7	224.94–304.73	282.32 ± 236.3^*^	223.14 ± 32.9^*^	246.86 ± 59.02	294.43 ± 64.35^*^	**<0.001**
CVMR_IES	382.34	336.52–448.65	383.59 ± 143.57	346.08 ± 81.36^*^	382.92 ± 66.29	447.78 ± 95.44^*^	**<0.001**
AST_IES	357.1	318.6–443.48	353.3 ± 88.68	317.8 ± 57.35^*^	351.86 ± 96.73	448.71 ± 141.53^*^	**<0.001**
IRT_IES	458.25	398.08–591.65	454.08 ± 187.52	394.6 ± 70.9^*^	469.82 ± 169.36	592.44 ± 221.69^*^	**<0.001**

* *If the variance of a variable differs significantly (p > 0.05) compared to other cases taken together, its Median ± SD is marked with an asterisk. The significant differences between cohorts are marked in bold*.

The results of Kruskal-Wallis test state that median values of the variables mentioned above differ significantly in the age groups. We also noticed that all groups are drawn from people with different median values of *AC* and *DMT* which is a marker of the information processing speed. However, there was no significant differences in the median values of *TRVI* between the groups (*p* = 0.148). The supposed reason for this is that TRVI values were highly scattered in the time scale in the group of adolescents (59.85 ± 60.46 ms). The values of the DMT are heteroscedastic throughout the lifespan, showing the high variance in adolescent group (63.08 ± 52.97 ms).

**Figure 3:**
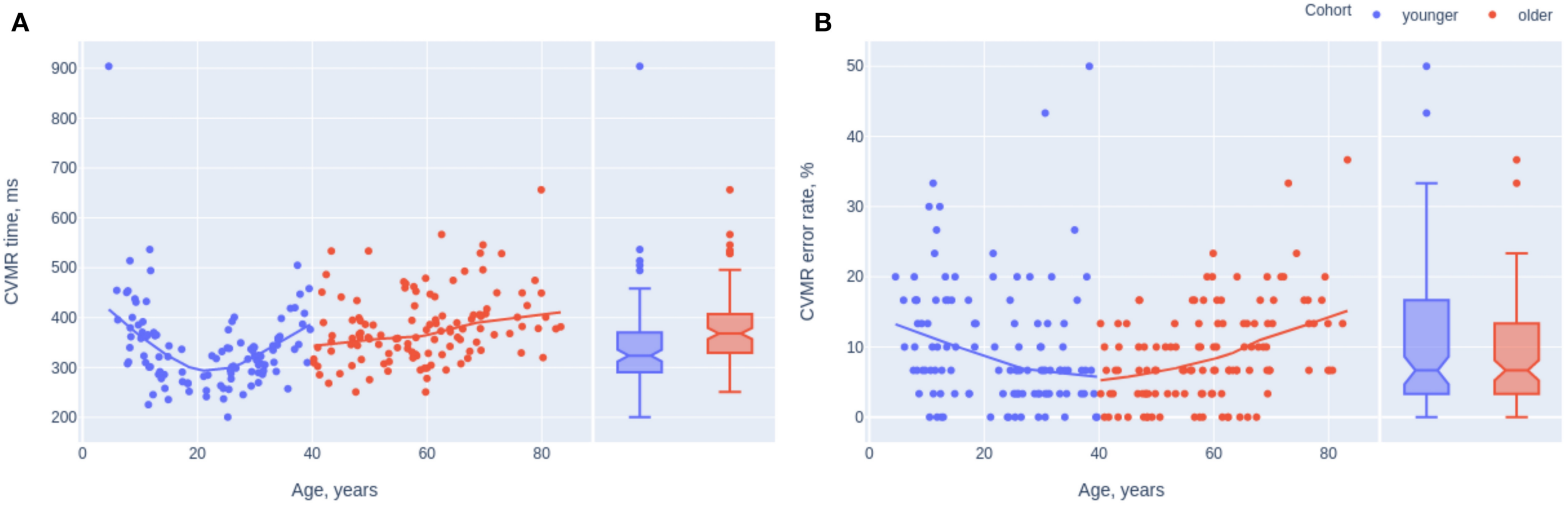
Scatterplots and trendlines presenting the dynamics of reaction time **(A)** and inaccuracy **(B)** while completing CVMR test. The participants below 40 years of age are marked in blue, above 40–in red.

Figure 3 describes age-related changes of mean RT and accuracy. To build the trendlines we used non-linear Locally Weighted Scatterplot Smoothing (LOWESS), which is a non-parametric regression method that combines multiple regression models in a k-nearest-neighbor-based meta-model.

### 4.2. The Association of Age and Cognitive Subdomains With the Novel IES From PT

#### 4.2.1. The Possible Associations Between the Age and the Newly Proposed Scores

**Figure 4:**
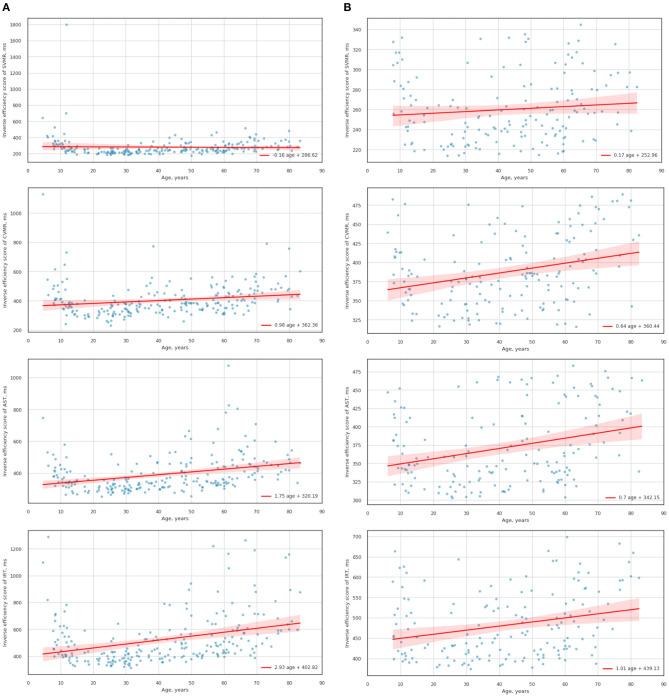
Scatterplots and trendlines of the lifelong changes in IES scores for psychophysiological tests (e.g., CVMR, SVMR, AST, IRT) before **(A)** and after removing the outliers **(B)**.

Figure 4A shows OLS regression trendlines with a 95% confidence interval. The trendlines reflect changes of SVMR_IES, CVMR_IES, AST_IES, IRT_IES throughout the lifespan. Figure 4B illustrates the age-related trends of the variables after the sensitivity analyses, in which the RT and error outliers are removed. It is worth mentioning that sample size was reduced to 161 points in average after the removal of the subjects which outstand the range from 15 to 85 percentiles. Table 2 presents the performance of the OLS regression models so that one can estimate how the data reproducibility improved with the outliers being removed.

**Table 2:** Characteristics of the linear models of the lifelong dynamics of IES for psychophysiological tests.

	**Whole dataset**				**Without outliers**			
	***SVMR_IES***	***CVMR_IES***	***AST_IES***	***IRT_IES***	***SVMR_IES***	***CVMR_IES***	***AST_IES***	***IRT_IES***
Slope	−0.159067	0.98327	1.74729	2.92665	0.16592	0.643205	0.7016	1.006818
Intercept	286.618	362.364	320.187	402.8234	252.9606	360.436189	342.1493	439.1339
*R*^2^	0.000741	0.040935	0.10976	0.108747	0.010645	0.083782	0.088361	0.06731
MAE	62.314987	69.338548	75.4813	128.482125	26.9876	35.992386	41.237137	66.063
MSE	15540.02	10314.252	11275.711	31964.0431	1075.506	1936.7576	2358.138119	6153.55

To compare the lifelong dynamics of the IES scores from different tests (e.g., CVMR, SVMR, AST, IRT) we analyzed the linear models. Specifically, we wanted to figure out if there is statistically significant differences between the variables' growth rate(slopes). For this we used statistical hypothesis tests, specifically *t*-test.

#### 4.2.2. The Association of the Novel IES for PT With the Cognitive Estimates

Table 3 reflects relationships between age, tests' results that reflect diverse cognitive subdomains and the proposed scores. In Table 4, there are Spearman rank correlation coefficients and the p-values that reflect the significancy of the associations of age with time estimates of switching conditions (e.g., DMT, TRVI).

**Table 3:** Association of the IES scores with the age and other psychophysiological tests results.

	***SVMR_IES***		***CVMR_IES***		***AST_IES***		***IRT_IES***	
	**Spearman**	***p*-value**	**Spearman**	***p*-value**	**Spearman**	***p*-value**	**Spearman**	***p*-value**
*Age*	0.178231	**0.00661**	0.309150	**<0.001**	0.375836	**<0.001**	0.385219	**<0.001**
*Error Rate, %*	0.431228	**<0.001**	0.300759	**<0.001**	0.507613	**<0.001**	0.446721	**<0.001**
*DMT*	−0.102335	0.12	0.463131	**<0.001**	0.121777	0.06	0.145347	**0.03**
*TRVI*	0.209057	**<0.001**	0.173764	**0.01**	−0.046755	0.48	0.461158	**<0.001**
*AC*	0.093113	0.15837	0.065160	0.32	0.141051	**0.03**	0.129164	*0.05*
*RMO_mean*	0.051543	0.44	0.079591	0.23	0.119649	0.07	0.053657	0.42
*RMO_variance*	0.587037	**<0.001**	0.548907	**<0.001**	0.581189	**<0.001**	0.562043	**0.01**
*RMO_mistakes*	0.543671	**<0.001**	0.517836	**<0.001**	0.533907	**<0.001**	0.590327	**<0.001**
*SVMR_variance*	0.744956	**<0.001**	0.700105	**<0.001**	0.507649	**<0.001**	0.514861	**<0.001**
*CVMR_variance*	0.490674	**<0.001**	0.674550	**<0.001**	0.440929	**<0.001**	0.446280	**<0.001**
*AST_variance*	0.567648	**<0.001**	0.541902	**<0.001**	0.833564	**<0.001**	0.651350	**<0.001**
*IRT_variance*	0.517957	**<0.001**	0.503068	**<0.001**	0.508932	**<0.001**	0.745864	**<0.001**

*The significant associations between features are marked in bold*.

**Table 4:** The association of the age and the time estimate of switching condition.

	**Age**	
	**Spearman**	***p*-value**
*DMT*	0.204	**0.002**
*TRVI*	0.077	0.242

### 4.3. Relationship Between the Mean Reaction Time and the Overall Number of Mistakes in Subcohorts

#### 4.3.1. The Age-Related Trends in the Reaction Time, Efficiency Score, and Mistakes

**Figure 5:**
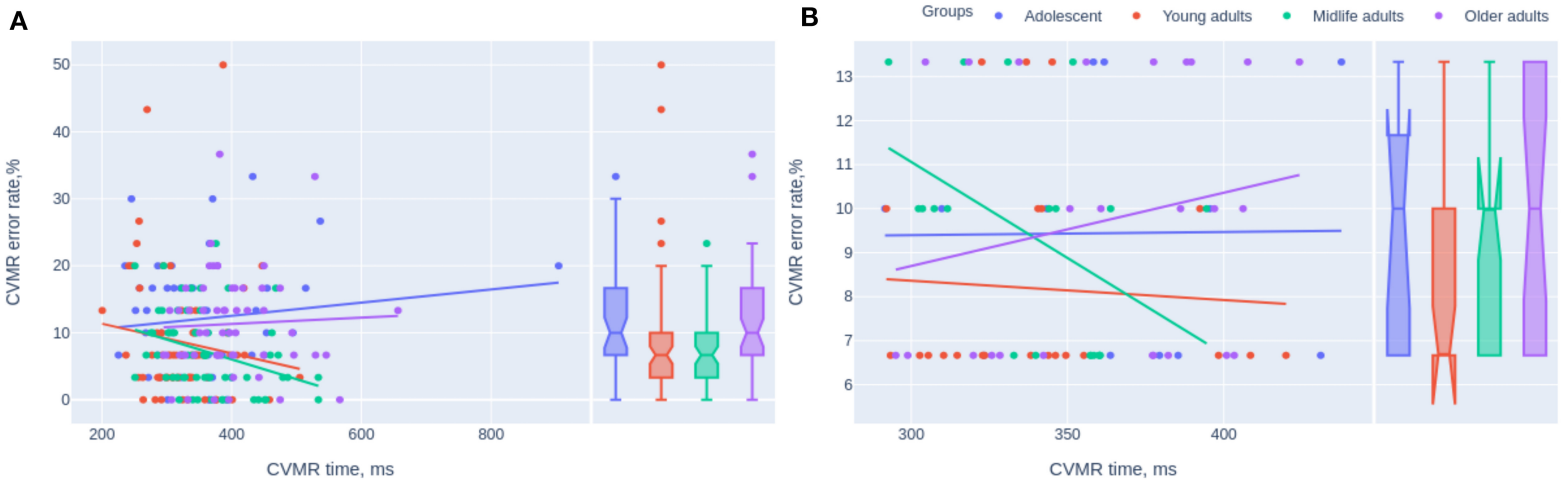
Scatterplots of the mean reaction time and the error rate in CVMR test. The trendlines present the tendencies for different age cohorts before **(A)** and after removing the outliers **(B)**.

To study the relationship between the mean reaction time and the overall number of mistakes in age-groups, we analyzed the OLS regression trendlines with a 95% confidence interval. Figure 5A presents the whole dataset whereas Figure 5B shows scatter plots with trends for the same groups after the sensitivity analysis. The removal of outliers for both variables (e.g., the reaction time and the number of mistakes) reduced the sample size down considerably to 79 cases. However, the observed tendencies remain roughly the same as before. With the reduced sample size it is hard to extrapolate them to the global population. Adolescents and older adults shared one tendency: the number of mistakes increased when the individual boosted the task completion. Young and midlife adults revealed the opposite tendency, i.e., the accuracy of their responses was better when the reaction was quick rather than when it was slow. Subsequently, we removed the outliers from CVMR time and CVMR error rate values which reduced the study sample size from 231 to 79 points. Nonetheless, the sample size reduction preserved the tendency when the people of the age range from 20 to 60 years (young and the midlife adults) had one type of time-to-accuracy association whilst adolescents and older adults had the opposite type. To check if there is an association between RT and age, ER we calculated Spearman rank correlation coefficients presented in Table 5.

**Table 5:** The association of the reaction time with the age and the percentage of the correct responses.

	**SVMR_mean**		**CVMR_mean**		**AST_mean**		**IRT_mean**	
	**Spearman**	***p*-value**	**Spearman**	***p*-value**	**Spearman**	***p*-value**	**Spearman**	***p*-value**
*Age*	0.219941	**0.00076**	0.317778	**<0.001**	0.368608	**<0.001**	0.382319	**<0.001**
*Error rate,%*	0.232336	**0.00037**	−0.057410	0.3851	0.515478	**<0.001**	0.524864	**<0.001**
*- in [20;60) years*	−0.11665	0.1897	−0.26003	**0.00303**	0.41222	**<0.001**	0.3593	**<0.001**
*- in <20 or ≥60 years*	0.3772	**<0.001**	0.01824	0.85489	0.56325	**<0.001**	0.6047	**<0.001**

*The significant associations between features are marked in bold*.

#### 4.3.2. The Gender-Related Traits in the Reaction Time, Efficiency Score, and Mistakes

Table 6 shows the descriptive statistics on the tests performance in both genders. At the bottom of the table it is well seen that females and males were distributed unequally across the age groups. There were significantly more boys rather than girls in the adolescent group (29.9 vs. 14.18%; *p* < 0.05) whilst the number of elderly women studied was significantly higher compared to men (29.85 vs. 15.46%).

**Table 6:** Characteristics of speed and accuracy performance in gender groups.

**Variable**	**Total**		**Female**	**Male**	**p_**2**__**−**__**3**_**
	***n***_****1****_ **= 231**		***n*_**2**_ = 134 (58.01%)**	***n*_**3**_ = 97 (41.99%)**	
Age	40.96	[24.87–59.76]	47.3 ± 20.08	33.73 ± 21.98	**<0.0007**
DMT	93.84	[63.6–122.43]	93.46 ± 58.5	95.18 ± 46.4	0.2835
TRVI	61.9	[34.7–96.0]	63.85 ± 58.98	59.1 ± 50.62	0.0767
AC	1.08	[1.01–1.19]	1.1 ± 0.19	1.04 ± 0.19	**<0.001**
SVMR_mean	245.52	[219.63–285.83]	249.55 ± 57.56	237.96 ± 61.31	**<0.0147**
CVMR_mean	350.75	[307.45–395.57]	356.04 ± 81.17	345.08 ± 77.4	**<0.0259**
AST_mean	343.4	[311.05–412.35]	357.9 ± 68.9	336.9 ± 59.13	**<0.0114**
IRT_mean	413.8	[368.15–471.85]	427.65 ± 77.99	401.8 ± 77.11	**<0.0021**
RMO_mean	7.6	[−18.5–31.35]	7.95 ± 91.16	7.6 ± 58.05	0.4155
SVMR_variance	57.7	[41.09–80.82]	57.18 ± 47.22	58.27 ± 48.48	0.3557
CVMR_variance	91.81	[70.7–118.64]	91.44 ± 79.9	96.42 ± 67.4	0.3801
AST_variance	72.0	[53.55–115.6]	76.3 ± 60.08	68.1 ± 46.35	*0.0533*
IRT_variance	103.5	[76.75–156.85]	107.7 ± 64.11	97.1 ± 67.76	0.4734
RMO_variance	140.3	[84.7–224.35]	161.3 ± 107.07	125.1 ± 95.13	**<0.0014**
SVMR_mistakes	0.0	[0.0–2.0]	0.0 ± 1.57	1.0 ± 2.91	0.1577
CVMR_mistakes	2.0	[1.0–4.0]	2.0 ± 2.49	3.0 ± 2.23	**<0.0003**
AST_mistakes	1.0	[0.0–2.0]	1.0 ± 2.77	0.0 ± 1.93	**<0.0467**
IRT_mistakes	3.0	[1.0–6.0]	3.0 ± 3.95	3.0 ± 3.44	0.1936
RMO_mistakes	22.0	[18.0–24.0]	22.5 ± 4.25	20.0 ± 4.83	**<0.001**
SVMR_IES	253.7	[224.94–304.73]	256.33 ± 72.12	249.57 ± 172.73	**<0.0451**
CVMR_IES	382.34	[336.52–448.65]	381.8 ± 110.89	383.04 ± 92.34	0.2482
AST_IES	357.1	[318.6–443.48]	368.6 ± 124.48	339.96 ± 89.24	**<0.0063**
IRT_IES	458.25	[398.08–591.65]	493.5 ± 191.49	433.14 ± 182.49	**<0.0036**
Adolescents	48 (20.78%)		19 (14.18%)^*^	29 (29.9%)^*^	**<0.0078**
Young adults	64 (27.71%)		36 (26.87%)	28 (28.87%)	
Midlife adults	64 (27.71%)		39 (29.1%)	25 (25.77%)	
Older adults	55 (23.81%)		40 (29.85%)^*^	15 (15.46%)^*^	

* *If the proportion of males and females in an age group is significantly different compared to other groups, such the group is marked with an asterisk. The significant differences between cohorts are marked in bold*.

**Figure 6:**
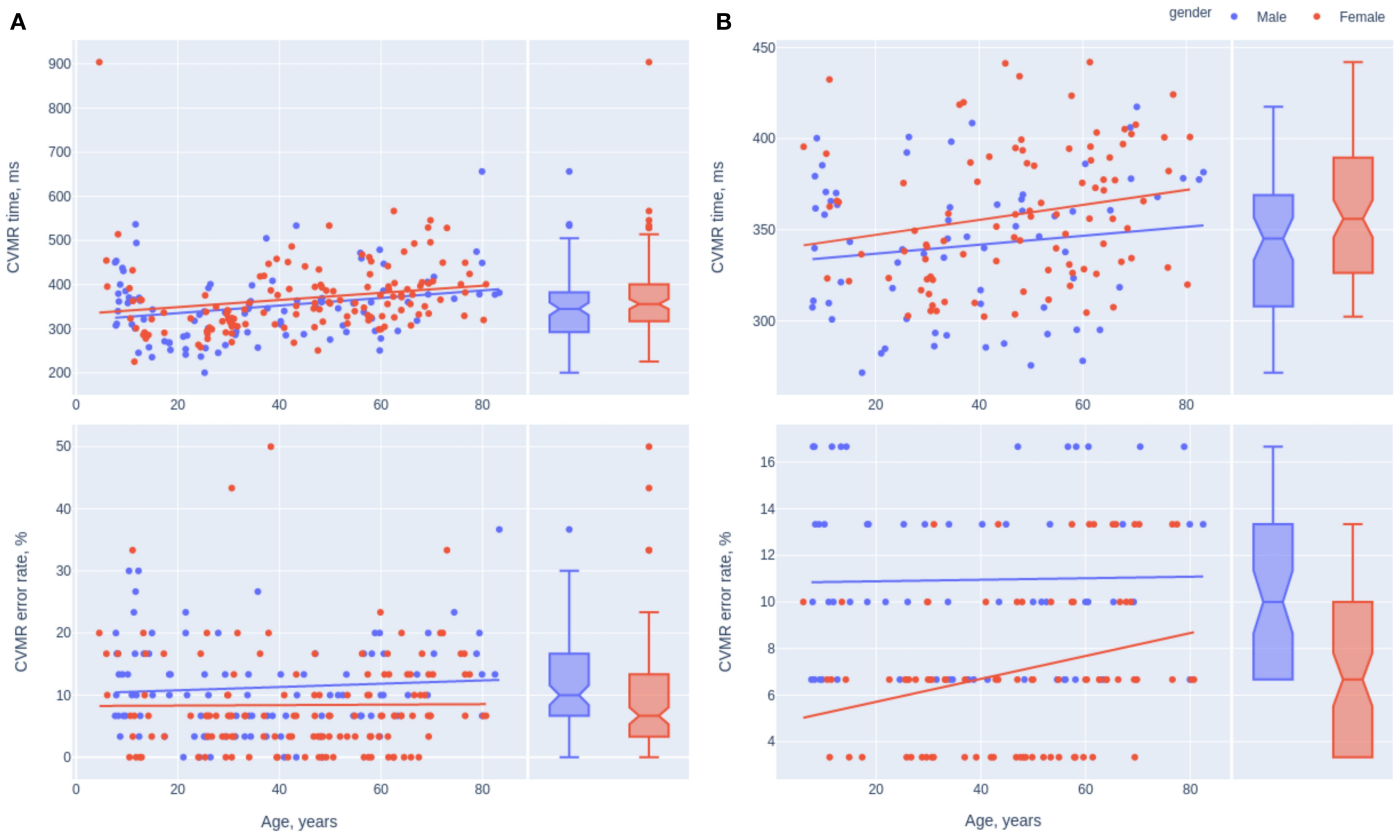
Scatterplots of the mean reaction time and the error rate in CVMR test. The trendlines present the tendencies for different gender with **(A)** and without the outliers **(B)**.

To perform the comparison we approximated the parameters of the model with the polynomial function of the degree one. We built the model for the overall study sample (Figure 6A) and for the values that are within the 15–85 percentile range separately (Figure 6B). The sample size decreased from 231 to 161 and 143 points for CVMR time and error rate, respectively.

Information on statistically significant differences between the intercepts and the slopes of the gender-specific models is in Table 7. Though both genders didn't show significant differences between reaction time speed decay throughout the lifespan, females seem to spend more time on the task on average, while performing it more accurately compared to men. After removing the outliers, the rate of the errors in females and males is significantly different (*p* = 0.00000866). Interestingly, males keep a stable accuracy level during the lifespan while the task performance accuracy drops with aging in the opposite gender.

**Table 7:** The interaction coefficients for the comparison of the intercepts and the slopes of the models.

		**A comparison of the intersepts**		**A comparison of the slopes**	
		**Estimate ± Std.Error**	***p*-value**	**Estimate ± Std.Error**	***p*-value**
*SVMR_IES* vs. *CVMR_IES*		75.74 ± 23.12	**0.00113**	1.1423 ± 0.4979	**0.02224**
*AST_IES* vs. *IRT_IES*		82.6360 ± 29.8992	**0.005943**	1.1794 ± 0.6440	0.067686
*SVMR_IES* vs. *AST_IES*		33.5681 ± 23.5458	0.154650	1.9064 ± 0.5071	**0.000193**
*CVMR_IES* vs. *IRT_IES*		40.4584 ± 29.5649	0.17184	1.9434 ± 0.6368	**0.00241**
**A cross-gender comparison**					
*CVMR_IES in females*	Full dataset	−17.7913 ± 29.4919	0.5469	0.3271 ± 0.6461	0.61302
vs *CVMR_IES in males*	Without outliers	24.97785 ± 16.0458	0.122	−0.5732 ± 0.3475	0.101
*CVMR_error_rate in females*	Full dataset	0.602601 ± 0.692298	0.385	0.006794 ± 0.015167	0.655
vs *CVMR_error_rate in males*	Without outliers	6.09494 ± 1.31926	**0.00000866**	−0.04594 ± 0.02785	0.1013
*CVMR_mean in females*	Full dataset	−14.48187 ± 22.57078	0.5218	0.04636 ± 0.49450	0.9254
vs *CVMR_mean in males*	Without outliers	−6.9771 ± 14.0149	0.6193	−0.1674 ± 0.2971	0.5739

*The significant differences between intercepts are marked in bold*.

### 4.4. The Predictive Potential of PTs to Identify the Values of Inverse Efficiency Scores

**Figure 7:**
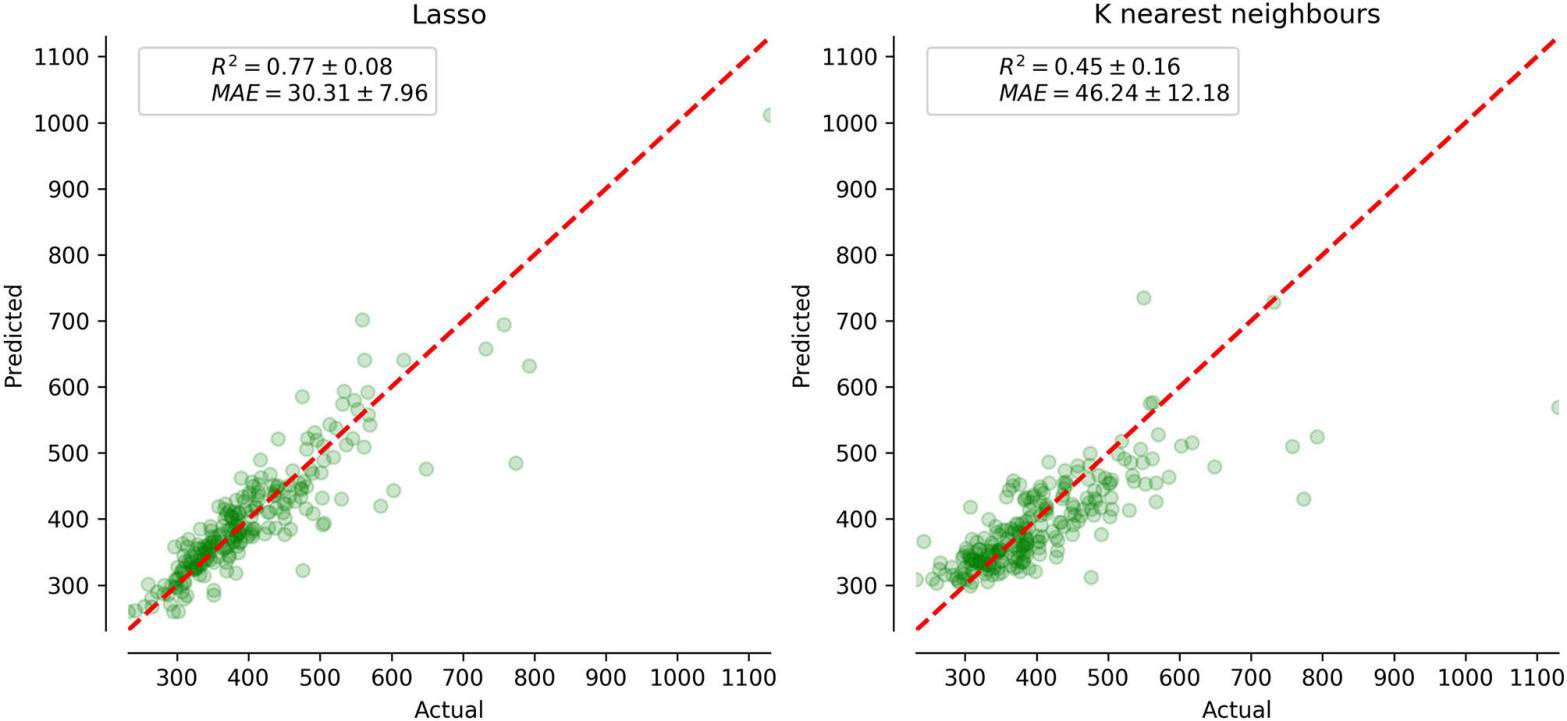
The performance of the employed IES prediction regression models and visualization of prediction error using 10-fold cross-validation technique on POBA datasets.

**Figure 8:**
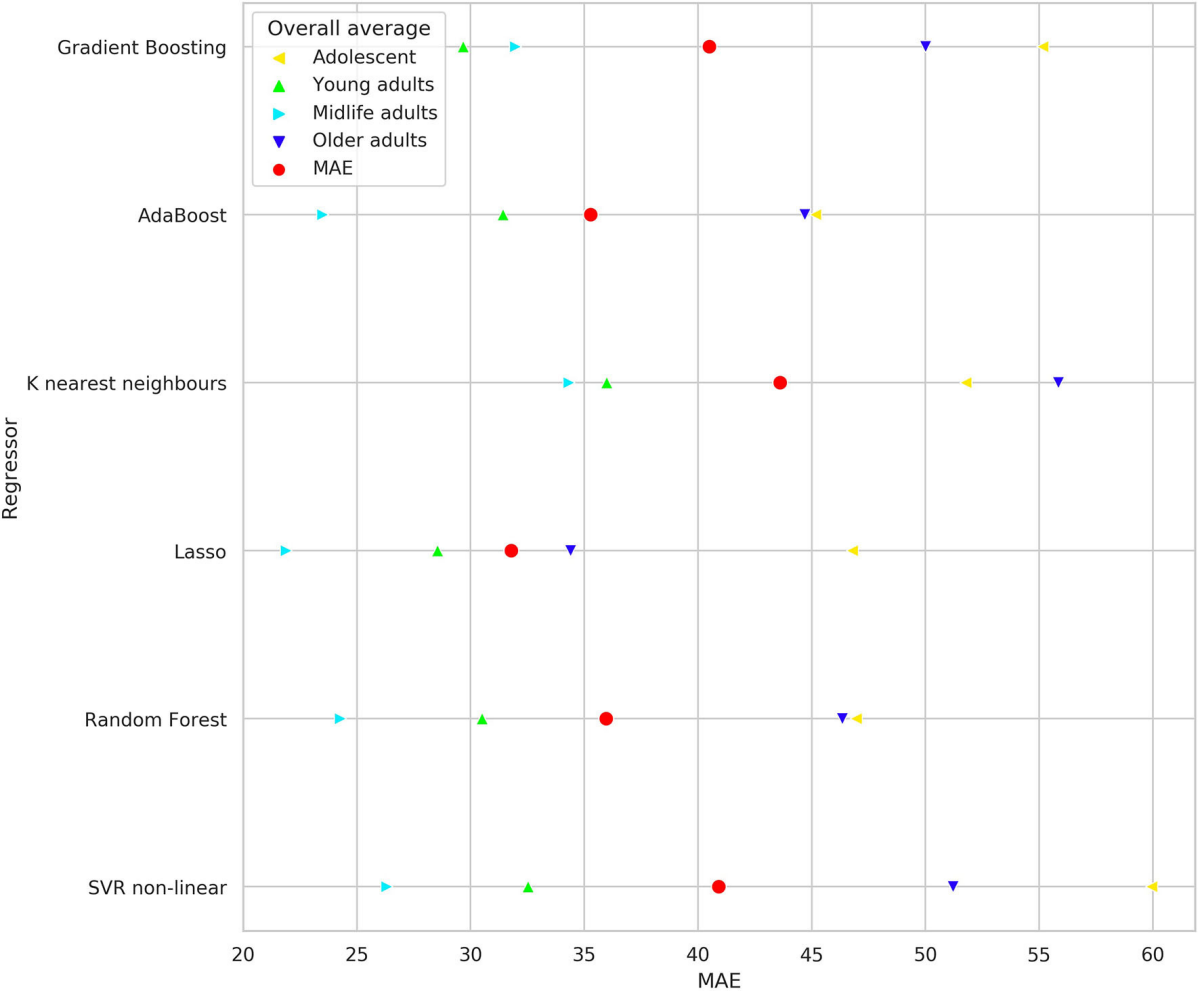
Mean absolute error of employed regression models for predicting IES in both datasets. The colored triangles mark the data calculated for age groups, the red circles indicate the value for entire dataset.

We built up a prediction model for the check-up if IES provides a summary of the findings obtained while testing individuals. The performance metrics of the regression model such as mean absolute error (MAE), root mean squared error (RMSE), and coefficient of determination (*R*^2^) are presented in Table 8. The graph in Figure 7 reveals the prediction error for IES in POBA dataset whereas Figure 8 presents the regression models' performance outcomes in different age groups.

**Table 8:** Performance metrics on CVMR_IES prediction.

**Regressor**	**MAE**	**RMSE**	***R*^2^**	**$\frac{MAE}{range\left(IES\right)},\%$**
Gradient boosting	36.372	55.549	0.652	4.05
AdaBoost	37.102	56.313	0.662	4.13
K nearest neighbors	46.241	70.768	0.447	5.15
Lasso	30.310	45.635	0.768	3.37
Random forest	37.227	58.216	0.653	4.14
SVR non-linear	39.484	63.278	0.590	4.39

#### 4.4.1. Studying the Association of the Performance Speed, Accuracy, and Stability

The trendlines from scatter plots in Figure 5 provide insight into the association of speed and accuracy of reaction. The examinees were set up neither for the time nor for the errorless behavior. This means they conducted the test with their regular performance. On the one hand, this does not allow us to calculate the speed-accuracy trade-off out of the data. On the other hand, such a paradigm is more useful to look for any age-related changes in the performance characteristics.

We tried to estimate if IES reflects the overall status of an individual. The battery of tests used in POBA describes individual psychophysiological status. We built up a regression model of IES prediction out of the PS data and estimate its performance metrics. They are presented in Table 8 and Figure 7.

## 5. Discussion

### 5.1. The Age-Related Changes of Reaction Speed and Performance Accuracy

Conducting the cross-sectional study we tried to find out if the slowing of the RT goes uniformly along with the decline in accuracy while aging. First, we studied the age-related changes in the time estimates of the tests (e.g., reaction time and decision-making time). From Kruskal-Wallis test (see Table 1), it is evident that the reaction time and its variance was relatively high in adolescents, then it reduced in adults and raised up in the remaining age groups. The accuracy performance had the opposite tendency: it was low in the adolescents, then it improved in the young adults and dropped back in the following years of life. We assume that adolescents experience more difficulties with the concentration of attention, and this impacts the performance of the attention-related tasks, particularly *AST* and *IRT*. Additionally, we tested the median values of the asymmetry coefficient and the tests results that reflect information processing speed such as *DMT, TRVI*. Interestingly, TRVI was the only variable the age-related samples of which originated from a common distribution. In other words, there is no significant difference in the median values of TRVI of the age groups. Meanwhile, DMT had age-related significant differences in median values. Out of this, we decided to concentrate on studying IES score for the CVMR task rather than for IRT task the derivate of which (e.g., TRVI) vary insignificantly across age groups.

After studying the time estimates we shifted our study to the lifelong dynamics of the accuracy (e.g., ER). One may expect the same lifelong dynamics of the accuracy of CVMR performance as in the reaction time. However, it is different (Figure 3). The number of mistakes made performing CVMR test reduces till the age of 40 and only then it starts rising. Conceptually, there is no way to delay the age-related structural changes while aging but there is a way to compensate them by using the biological reserves. Moreover, the concept of reserve can be easily applied to brain aging. For example, the reaction time may slow with age, while knowledge of world events may expand. According to Cullati et al. (2018), cognitive reserve is seen as actively acquired, and stimulating activities, like educational achievement over the life course, result in cognitive reserve maintaining or improvement. To test the hypothesis if the continuing education accounts for the decrease in the number of mistakes we may potentially use such proxies as the total number of years of education. However, they do not serve as the direct measure of intellectual functioning. It must be mentioned that participants at the age range of 40–50 years had the best test performance in terms of response accuracy. The error rate was <15% in almost all the cases. For this level of accuracy authors tend to analyze RT rather than ER, especially in case of the confusive findings when both RT and ER point in the same direction (Bruyer and Brysbaert, 2011). Furthermore, Akhtar and Enns (1989) suggest that IES better not used when ER >10%. There is no consensus on what is the threshold value of ER when researchers can rely on IES. Because of this, a study should not be limited to the analysis of the IES score. Moreover, the information on RT is not limited to its absolute value, the variance of RT across trials reflects the reaction stability. Such information is totally missing in IES.

### 5.2. The Association of the Proposed Efficiency Scores With the Age and Cognition

In total, the statements about the data encompassing the POBA dataset and the assumptions that come from their analysis is consistent with the concept of speed-accuracy trade-off and its age-related changes. Studying the lifelong tendencies of the tests (see Figure 4), we see that the IES scores for all the PT increase steadily with age except the SVMR_IES. Notably, the values of the reaction time for SVMR were scattered more than for other tests. This may result from the study methodology. We started testing people with the SVMR test as the easiest one. Although it is a simple one, some experience is required to get used to it. This assumption is in line with a formal model of decision making by Stone (1960) which supports the fact of the information accumulation over the course of perceptual decision-making (Stone, 1960; Heitz, 2014). It is common sense that adolescents if compared to other age groups have the lowest number of the total years of education. The lower education level and the attention deficit typical of this age may result in the rate of adoption to the tasks we delivered. Because of this, the variance for reaction time for SVMR in adolescents was bigger than its mean value (SVMR_variance > SVMR_mean) unlike for the other tests (e.g., CVMR_variance < CVMR_mean, AST_variance < AST_mean, IRT_variance < IRT_mean). After removing the outliers, the negative trend for the lifelong dynamics of SVMR_IES was reversed. However, this did not change the age-related tendencies in the other tests (e.g., CVMR, AST, IRT). The significant reduction of the sample size makes it less representative and may hide tendencies that were remarkable in the full dataset. However, the removal of the outliers is required for the analysis of the lifelong changes in SVMR metrics. From Table 2, the age prediction out of the AST_IES and IRT_IES values has better performance if the model is trained with the full dataset rather than without them (e.g., *R*^2^ = 0.10976 vs. 0.088361 for AST; 0.108747 vs. 0.06731 for IRT). To sum up, IES for PT undergo age-related changes that can be described as the neurocognitive slowing, i.e., the latencies increase steadily. We may consider IES as markers of the brain aging because they incorporate information both on RT and accuracy. We also showed the ambiguity of the removal of the dataset outliers for the reproducibility of the data.

As the battery of PT provides a set of time and accuracy estimates, several IES scores derive from diverse task paradigms. This rises up a question of the comparison of the scores and their association with the age. On Figure 4B it is well seen that the rate of age-related IES progression increased with the difficulty of the task (see the slopes values in Table 2). As seen from Table 7, there is a significant difference in the slope value in SVMR_IES compared to CVMR_IES (*p* = 0.02224). This is in consistency with the other researchers who found a difference in the time for processing the simple reaction task (0.5 ms/yr) vs. the complex one (1.6 ms/yr) (Fozard et al., 1994). At the same time, it is hard to explain why the slopes for AST and IRT do not differ significantly (*p* = 0.067686). The interfering objects in IRT test add the switching condition which is absent in AST similarly like CVMR has a switching condition while SVMR does not. Because of the switching condition we expected that the lifelong dynamics of IRT should differ significantly from AST. As it was not so, we decided to proceed with the exploration of the CVMR_IES, its age-related trends, and gender-specific features.

Another way of comparing the proposed IES for PT is by using the cognitive approach. We looked for the score that has the strongest informative power to reflect changes in cognitive subdomains. From Table 3, the association of the psychophysiological estimates of the information processing speed (DMT, TRVI) with CVMR_IES is more tight (*p* ≤ 0.001) than with IRT_IES (*p*-value p to 0.03). This was another reason for us to decide to preserve CVMR_IES as the major marker of the speed-accuracy performance for the battery of tests.

Thus, it looks promising to propose IES for PT to cognitive studies in aging neuroscience for a set of reasons. First, there is an evident dynamics of the indices throughout the lifespan. Second, neurocognitive slowing estimated with IES-like indices correlates with task difficulty. Finally, the indices are reflective of cognitive subdomains (e.g. switching and inhibition).

### 5.3. The Relationship Between the Reaction Time and Accuracy

#### 5.3.1. Age-Related Aspects

The concept of SAT implies the presence of negative correlation between RT and ER. However, many authors use IES without the preliminary checkout if there is a negative correlation between RT and ER. In our dataset, the “adolescents” and the “older adults” shared one tendency regarding the speed-accuracy ratio without SAT, while the other two groups shared the opposite trend (see Figure 5A). Both at the time of active developmental changes *in adolescence* and during ongoing atrophic changes *in elderly* there is the tendency toward the rise of the number of mistakes while slowing the reaction. The quicker the task is done the better the accuracy is or vice versa. We hypothesize that there are two options when we talk about an 'older adult: the person is either cognitively preserved and has a good task performance or the individual is somehow cognitively impaired, which reduces all metrics of performance (e.g., both the reaction time and the reaction accuracy). The examinees in the age range from 20 to 60 show the opposite relationship between the speed and accuracy. Slow reaction (long reaction time) is associated with the low number of mistakes which makes sense for the regular condition of an adult. So, in this age group we see the presence of SAT. In our battery of tests CVMR was the only one with the significant negative association between RT and ER in the subcohorts of young and midlife adults taken together (see Table 5). This goes in line with a study of Townsend and Ashby who advised to use IES only in case of a high and linear correlation between RT and ER (Townsend and Ashby, 1978; Townsend et al., 1983). However, an experiment by Ferrand evidenced that a positive correlation between RT and ER does not necessarily mean that more variance will be explained better in the IES rather than in RT measures (Ferrand et al., 2010; Bruyer and Brysbaert, 2011). This is exactly what we see in Tables 3, 5, i.e., the variance of ER is explained better in the CVMR_IES (*r* = 0.300759; *p* < 0.001) than in CVMR_mean (*r* = −0.057410; *p* = 0.38). Many researchers do not follow the recommendations of Townsend and Ashby to check if RT and PE are positively correlated before using IES (Goffaux et al., 2005; Jacques and Rossion, 2007; Kuefner et al., 2010). Some authors do not analyze PE and ER but go only for the analysis of IES (Minnebusch et al., 2008). Other authors report SAT as an issue which rises up when the examinee is set up for either time or errorless performance (Murphy and Klein, 1998; Kennett et al., 2001). But there is no common solution accepted by the international research society.

#### 5.3.2. Gender-Related Aspects

One may expect that psychological traits of men and women result in different age-related dynamics of RT or ER. The age-related changes of the task performance look similar in both gender disregarding the fact whether we analyze the total study cohort (Figure 6A) or the reduced sample after removing the outliers (Figure 6B). In general, the rate of both the neurocognitive slowing and the accuracy reduction is more pronounced in men rather than in women. In both samples with or without outliers we observed insignificant advantage in RT for male participants. In part this reproduces the results of a study by Adam (1999) who also found a near-significant overall reaction time advantage for male participants. The authors suggested that gender difference in reaction time performance may reflect differences in processing strategy. Other researcher achieved confusing results that there was no significant difference in RT between people of both gender during a unimanual speed-accuracy task, however during a bimanual task, the reaction time of both hands was significantly longer in women than men (Mickevičienė et al., 2011). We didn't find significant differences in the slopes of the linear models describing reaction time and error rate (see Table 7). After removing the outliers, the average number of mistakes was significantly higher in males than in females. The results stay in line with the data from Larson et al. that females and subjects high on neuroticism made significantly fewer errors in a choice reaction-time task (Larson and Saccuzzo, 1986). In an experiment with the bimanual task the accuracy of the left hand was significantly greater in men than women (Mickevičienė et al., 2011). Thus, we can report the presence of some gender-dependent features of task performance. According to our data and some references (Dane and Erzurumluoglu, 2003; Barral and Debû, 2004), women perform more slowly and accurately than men in the speed-accuracy task.

### 5.4. The Informative Value of the Inverse Efficiency Score in PT

Hypothetically, IES may serve as a marker of psychophysiological status. If so, machine learning algorithms should be able to predict it out other PT results. Table 8 illustrates the predictive potential of PTs to identify the values of inverse efficiency scores. The performance metrics are good: the proportion of MAE to the range of IES values is low for all regressors (3.37–5.15%). Lasso regressor has the best performance, the *R*^2^ value for it is close to 1.0, and this justifies the high accuracy of the regression model. The graph in Figure 7 visually evidences low prediction error for IES in POBA dataset. The regressors with the best (Lasso) and the worst (K nearest neighbors) are shown. Visually, the difference between them is insignificant, i.e., all the created regression models are quite reliable. Figure 8 presents the prediction performance of the regression models; the performance varies regarding the age. MAE is maximal for adolescence and a bit lower for older adults group. We can explain this by the individual rate of cognitive changes at the beginning and at the end of life. This assumption looks convincing cause neurodevelopment and aging are highly individual processes. In POBA dataset, all the models provide the best prediction for young and middle-aged adults. This supports the presence of SAT in the age groups mentioned above and the applicability of IES to them.

Developing the IES for clinical use may improve the currently existing strategies for the early diagnostics of dementia. The supposed benefits are the following. First, the application of the tests with SAT will provide physicians and clinical psychologists quantitative metrics of the cognitive status. The quantification is crucial for follow-up studies. Second, deploying machine learning algorithms for predicting biological age may help to organize the population screening for dementia to find suspicious cases at an early stage. The tasks that include the SAT condition seem more informative because they may reflect cognitive impairment in a set of domains of executive functioning. To make the screening more proficient, we suppose to utilize the machine learning approach for fusing the evidence from such psychophysiological tests with the one that comes from radiological findings (e.g., brain MRI).

## 6. Limitation of the Study

The known limitation of the study lies in the speed-accuracy ratio and payoff conception itself. It turns out that the assumption on the relative time cost of correct decision works for the summary statistics of the overall results obtained throughout the entire test. But if analyzed separately, the average reaction time for the error responses is faster than that for the correct ones.

Moreover, the time cost of making an error depends on the level of accuracy at which one is operating at the moment. An error saves more time at high levels of accuracy than at low levels. This means that the initial intent to figure out the time cost of making an error does not make sense. It is rather meant to estimate how long it requires to improve the confidence of the confidence of the examinee's judgment by a factor of two or ten compared to the present level of performance.

## 7. Conclusion

- IES score is potentially a clinically useful metric for summarizing the overall efficiency of decision making. It can be a particularly reliable tool when applied to psychophysiological test results as it reflects speed-accuracy performance and age-related changes. As a dependent variable for the complex visual-motor reaction test, IES is the best out of four newly proposed indices. IES accounts for different cognitive subdomains and may reflect their disproportional changes throughout the lifespan. This encourages us to explore psychophysiological test results utilizing machine learning models that can be designed as a reliable computer-aided detector of cognitive changes at an early stage.
- The examinees under 20 and over 60 years of age share one tendency regarding the speed-accuracy ratio without speed-accuracy trade-off. Both at the time of active developmental changes in adolescence and during ongoing atrophic changes in elderly there is a tendency toward a rise of the number of mistakes while slowing the reaction. In the age range from 20 to 60 the relationship between the speed and accuracy is opposite. In our battery, complex visual-motor reaction is the only test with the significant negative association between reaction time and error rate in the subcohort of young and midlife adults taken together.
- On average, women perform more slowly and accurately than men in the speed-accuracy task, however most of the gender-related differences are insignificant. Interestingly, males keep a stable accuracy level during the lifespan while the task performance accuracy drops with aging in the opposite gender.
- The IES index for PT is a reliable index that describes the individual psychophysiological status. Using results of other psychophysiological tests, we predicted IES values for the visual-motor reaction with high accuracy (*R*^2^= 0.77 ± 0.08; mean absolute error/IES range = 3.37%). The regression model shows the best performance in the cognitively preserved population groups of young and middle-aged adults (20–60 yrs). The individual rate of neurodevelopment in youth and atrophy in elderly results in worse prediction performance of the regression model.

## Data Availability Statement

The datasets presented in this study can be found in online repositories. The POBA dataset generated for this study can be found at the site of Data Analytics Group at: https://bi-dac.com.

## Ethics Statement

The studies involving human participants were reviewed and approved by United Arab Emirates University Human Research Ethics Committee. Written informed consent to participate in this study was provided by the participants' legal guardian/next of kin.

## Author Contributions

YS and TH contributed to the conceptual idea of the paper. YS formulated the objectives and wrote the manuscript. TH performed the statistical analysis, prepared the figures and tables for data presentation and illustration. KG with NZ and TA contributed to the literature review and data analysis. All authors contributed to the article and approved the submitted version.

## Conflict of Interest

The authors declare that the research was conducted in the absence of any commercial or financial relationships that could be construed as a potential conflict of interest.

## Abbreviations

AC: asymmetry coefficient
AST: attention study technique
CVMR: complex visual-motor reaction
DMT: decision making time
ER: error rate
IES: inverse efficiency score
IQR: interquartile range
IRT: interference resilience technique
LOWESS: locally weighted scatterplot smoothing
MAE: mean absolute error
ML: machine learning
OLS: ordinary least squares
POBA: psychophysiological outcomes of brain atrophy (the name of a project and the dataset obtained)
PS: psychophysiological status
PT: psychophysiological test
RMO: reaction to a moving object
RMSE: root mean squared error
RT: reaction time
SAT: speed-accuracy trade-off
SVMR: simple visual-motor reaction
TRVI: the time delay in responding to the targeted stimulus because of visual interfering objects.
